# Agreement between Two Devices for Measuring Pupil Diameter in Patients Implanted with Multifocal Intraocular Lenses

**DOI:** 10.3390/vision7020040

**Published:** 2023-05-09

**Authors:** Joaquín Fernández, Noemí Burguera, Carlos Rocha-de-Lossada, Marina Rodríguez-Calvo-de-Mora, Manuel Rodríguez-Vallejo

**Affiliations:** 1 Department of Ophthalmology (Qvision), VITHAS Almería Hospital, 04120 Almería, Spain; 2 Department of Ophthalmology, VITHAS Málaga, 29016 Málaga, Spain; 3 Department of Ophthalmology, Hospital Regional Universitario de Málaga, Plaza del Hospital Civil, S/N, 29009 Málaga, Spain; 4 Departamento de Cirugía, Universidad de Sevilla, Área de Oftalmología, Doctor Fedriani, S/N, 41009 Sevilla, Spain

**Keywords:** pupil diameter, photopic, mesopic, average, multifocal intraocular lenses

## Abstract

The purpose of this study was to evaluate the agreement between three methods for measuring pupil size in patients implanted with multifocal intraocular lenses (MIOLs): Keratograph 5M (K5M), Pentacam AXL Wave (PW), and a simple hand ruler. Sixty-nine subjects implanted with MIOLs and measured at the three-month follow-up visit were included in this retrospective analysis. K5M and PW were used to measure the photopic (PP) and mesopic (MP) pupil sizes, and a hand ruler was used to measure the pupil under environmental light conditions (135 lux). The Bland–Altman method with its limits (LoAs) was used to assess the agreement. The median PP was 2.8, 2.95, and 3 mm for K5M, PW, and the ruler, respectively (*p* < 0.05). Differences in PP were statistically significant for all paired comparisons (*p* < 0.0005) except between PW and the ruler (*p* = 0.44). The LoAs for the difference in PP between K5M and PW was 0.63 mm. The mean difference for MP between K5M and PW was 0.04 mm (*p* = 0.34) with LoAs of 0.72 mm. MP measured with K5M and PW could be considered interchangeable, although a correction of −0.3 mm (IC95%: −0.23 to −0.39) should be applied to PP measured with PW to attain the K5M mean.

## 1. Introduction

The pupil diameter is a mandatory measurement in screening procedures for cataract surgery and refractive lens exchange with the implantation of a multifocal intraocular lens (MIOL). The importance of this measurement is justified by the variations in visual performance in patients implanted with MIOLs depending on pupil size [1,2,3] which could lead to the selection of a particular design depending on the patient’s pupil [4]. Historically, this measurement has been conducted with devices known as pupillometers, which are specially designed for this purpose, such as the Colvard (Oasis Medical, Glendora, CA, USA) [5]. However, new devices such as videokeratographers, tomographers, and aberrometers incorporate this measurement as a complementary tool beyond the main measurement purpose [6,7]. These devices can use flashlights with different intensities to measure the Photopic Pupil Diameter (PP), while Mesopic Pupil Diameter (MP) can depend on the environmental light conditions in which the measurement is taken. Therefore, the knowledge of the device being used for measuring pupil diameter and the use of this device under appropriate environmental light conditions are mandatory for interpreting its possible relationship with visual performance.

Beyond the previously-described technologies, the PP can be also measured, though less precisely, with a simple ruler [8]. However, this requires infrared light to evaluate the latter in mesopic (MP) conditions. This assessment, despite its simplicity, could be more highly correlated with visual performance if it is conducted in the same environmental light condition where visual acuity is tested [9]. Interpretation of MIOL studies reporting visual performance changes with pupil diameter could be quite difficult considering the differences in the testing conditions. It is important to note that pupil size depends on environmental light intensity, and this environmental light usually depends on the patient’s activities. For instance, reading tasks require a range between 300 lux and 500 lux in places such as offices, libraries, and exam lanes where eye examinations are conducted [10]. This range of light intensities is consistent with the flashlights used by several devices, such as Keratograph (K5M) and Pentacam (both by Oculus Optikgeräte, Wetzlar, Germany). However, very demanding tasks at near vision, such as working with precious stones, require even higher levels of light intensity (1500 lux) [10]. Therefore, some questions that the surgeon should consider are: What are the tasks conducted by the patient (working distances)? What is the environmental light level at which the patient conducts these tasks? And finally, what is the visual performance achieved with the MIOL considering the pupil diameter for these distances and environmental lights? Obviously, the final answer that will help us to select the MIOL requires much knowledge of the pupil dependency of the optical design, but also, as importantly, how the device being used measures the pupil diameter.

The distribution of PP and MP in patients implanted with MIOLs has been previously published for the K5M [11], which is a videokeratographer that dynamically allows the measurement of pupil diameter. However, this device is not commonly used to report pupil diameter in clinical studies of MIOLs [12,13]. Pentacam AXL, a tomographer and biometer, is more commonly used by anterior segment surgeons due to its versatility, incorporating the measurement of the static MP in its latest Wave version (PW). Previous clinical studies on MIOLs have used this device to report PP. For instance, Tañá-Sanz et al. [14] reported a mean pupil size of 2.4 mm in a sample of patients with a mean age of 72 years, and Sun et al. [15] reported a mean pupil size of 3.3 mm in a sample of patients with a mean age of 66 years. Other authors have also used Pentacam for reporting PP in their studies with mean pupil sizes in the range of 2.4 to 3.3 mm depending on the mean age of the sample [16,17,18,19]. As PW now measures MP, it is expected that future studies will also include the MP with this device; therefore, it is important to know how the measurement agrees with other previous devices such as the K5M. The aim of this study was to evaluate the agreement between the K5M, PW, and a simple ruler for measuring pupil diameter. The results of this study are of importance for the screening criteria selection of MIOLs using the pupil diameter of current PW users who previously used K5M to conduct these measurements.

## 2. Materials and Methods

### 2.1. Subjects

This retrospective study was approved by the Ethics Committee of Research, Almería Center, Torrecardenas Hospital Complex, and adhered to the tenets of the Declaration of Helsinki. A total of 69 eyes and subjects consecutively implanted with the Liberty (Medicontur Medical Engineering Ltd., Inc., Budapest, Hungary) from February 2021 to January 2022 at Qvision, Ophthalmology Department (VITHAS Almería) whose pupil diameters were measured during the 3-month follow-up visit with K5M, PW, and a hand ruler were included in the study. The exclusion criteria were those for which an MIOL implantation was not recommended according to our current standard clinical practice: any disease affecting visual acuity and high-order aberrations above 0.5 microns, measured with PW at 4 mm.

### 2.2. Measurement Procedures

The pupil size of the subjects was measured using our current patient journey map. First, pupil size was measured using K5M followed by PW in a different room under mesopic conditions, approximately 5 lux, by the same technician. The K5M is a multipurpose system that includes a Placido disk corneal topographer and a wide-field camera. This device uses infrared light and a video camera that dynamically allows for the measurement of pupil size with the response to consecutive flash lights of 0.2 s followed by a brief mesopic adaptation of 9.8 s [11]. The PP and MP were obtained by averaging the results obtained after three consecutive measurements. Although this is a multipurpose system, only the “Pupillogram” automated mode was used.

Patients were moved to the PW room and a single full-sequence measurement was conducted approximately 3 min after K5M measurement completion. The PW is a combined optical biometer (partial low-coherence interferometry), tomographer (Scheimpflug), and aberrometer (Hartman–Shack). The full-sequence procedure was conducted in all eyes with this device, starting with the wavefront measurement, where the static MP is captured in infrared light, followed by a retro-illumination capture through the pupil, the measurement of axial length, and finishing with tomography, where PP is measured through the response to a rotation slit light scan. The last measurement of PP with a ruler was carried out in an exam lane with an environmental light of 135 lux while the patient observed an ETDRS chart located at 4 m, which was measured prior to the common ophthalmologic examination procedures such as refraction and visual acuity.

### 2.3. Statistical Analysis

Although both eyes were consecutively measured, starting with the right eye and followed by the left eye, only a random eye was included in the study due to high within-eye intraclass correlation coefficients, >0.8 in all the comparisons, except for the MP measured with PW which was 0.67 (*p* < 0.0005). Normal data distributions were confirmed using the Kolmogorov–Smirnov test. Randomization was conducted using a personalized random function created in MATLAB (R2019a; MathWorks, Natick, MA, USA). Any error in systematically measuring the left eye after the right eye was also discarded, obtaining differences below 0.06 mm between eyes in all cases (*p* > 0.05), except for the MP with PW that was 0.14 mm higher with the right eye (*p* = 0.12), tested with a *t*-test for paired samples. Differences between pupil diameter measurement procedures were analyzed using the Friedman test with Bonferroni correction for post hoc comparison when the PP measured with the ruler (non-normally distributed) was included in the analysis, with the paired *t*-test for the comparison of MP with K5M and PW, and the independent *t*-test for testing differences between men and women. Data analysis was performed using the IBM SPSS for Windows statistical software (version 24.0; SPSS, Inc., Chicago, IL, USA). Agreement was assessed by computing the Bland–Altman plots using Carkeet Excel file and 2-sided tolerance factor for computing 95% confidence intervals [20].

## 3. Results

A sample of 29 men and 40 women with a mean age of 64 ± 9 years was included in the analysis. No significant differences in age were obtained between sex groups (*p* = 0.79). However, men had smaller PP and MP than women, −0.33 mm (*p* = 0.02) and −0.53 mm (*p* = 0.007), respectively, for K5M, whereas for PW, mean differences of −0.37 mm (*p* = 0.005) and −0.51 mm (*p* = 0.008) were obtained, respectively. These smaller pupil sizes for men were not as obvious in the measurement with the ruler at −0.12 mm for PP (*p* = 0.28).

Table 1 shows the descriptive statistics for pupil diameter measurements for each procedure. Differences were found between K5M and PW and the ruler (*p* < 0.0005) for PP but not between PW and the ruler (*p* = 0.44). No differences were found between K5M and PW for MP (*p* = 0.34).

**Figure 1:**
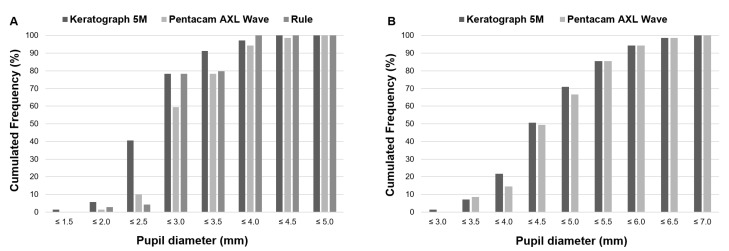
Cumulative percentage of eyes achieving a particular pupil diameter with each device in (**A**) photopic or (**B**) mesopic pupil diameter.

Figure 1A shows these differences between methods for PP, more remarkably for pupil diameters ≤ 2.5 mm, where the cumulative percentage of eyes was 40.6% for K5M, whereas PW and the ruler only achieved 10.1% and 4.3%, respectively. On the other hand, nearly 80% of eyes resulted in a PP smaller than 3.5 mm and almost 100% were below 4.0 mm. Cumulated percentages of eyes for MP are described in Figure 1B. The MP was very uniform between both methods of measurement.

**Figure 2:**
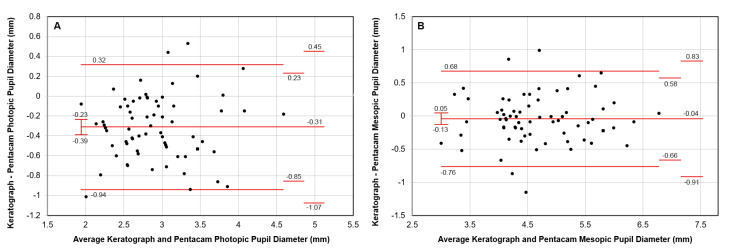
Agreement between Keratograph and Pentacam AXL Wave in the measurement of the (**A**) photopic and (**B**) mesopic pupil diameter. Exact 95% confidence intervals are shown above and below the mean difference and limits of agreement.

Figure 2 shows the agreement between K5M and PW for PP with a mean difference of 0.3 mm smaller diameter for K5M. The LoAs at 1.96 standard deviations from the mean were narrower for the PP (0.63 mm) than for the MP (0.72 mm).

## 4. Discussion

Pupil diameter is a key measurement in the preoperative screening for the selection of a MIOL, as visual performance may vary with this parameter and patient satisfaction can be affected by pupil size [1,2,3,21]. Beyond the question of which MIOL design is selected depending on this preoperative measurement, other questions may arise such as which is the minimum or maximum pupil size at which a particular MIOL design should be discarded. To answer these questions, clinical studies should report the visual performance stratified by pupil size [22]. However, this information would have little or no value if the clinician is using another device for measuring the pupil diameter, and a previous agreement study has not been conducted to confirm that results could be interchangeable. In this study, we evaluated the agreement between two devices: the K5M, for which pupil diameter distribution with MIOLs has been previously published [11], and the PW, a device that integrates a wide number of measurements required for preoperative screening. Furthermore, the agreement of the PP with the measurement using a hand ruler was analyzed for both devices.

Our results for K5M and PP were in agreement with the results published in a previous study by our research group, 78.3% vs. 84.5% of eyes ≤ 3 mm and 91.3% vs. 95.8% of eyes ≤ 3.5 mm [11]. However, it is important to note that these percentages for smaller PP were not in agreement with the PW, which showed percentages of 59.4% and 79.3% for these diameters, respectively. On the other hand, all devices showed PP below 4 mm in nearly 100% of eyes, which means that an MIOL that provides near and intermediate vision inside of patient expectations at 4 mm will be able to be generally selected independently of the loss of intermediate/near vision above this pupil diameter. Moreover, the K5M and PW provided similar outcomes for MP due to this measurement being obtained at the environmental lighting conditions. The percentage of eyes with MP ≤ 5 mm was lower in the current study than in the previous one [11], 71% versus 93.3%. The role of MP in the selection of MIOL differs from the role of PP, while the PP is related to intermediate and near vision tasks for which high light conditions are required, the MP is related to the loss of visual quality due to the spherical aberration (SA) which can be compensated or increased by the SA induced by the MIOL. The importance of SA in MIOL selection has offered controversial results among studies [23,24,25,26]; this is mainly due to devices usually providing the SA at 6 mm and this should be recalculated for the mesopic pupil size. While the SA also could have some influence on the photic phenomena size, the relationship between MP and photic phenomena is driven by the addition and extension of rings in diffractive MIOLs [27]. Therefore, the surgeon should make a decision considering a balance between maintaining intermediate and near vision by taking into account the PP, and minimizing the increase of photic phenomena by considering the MP.

Before new devices for measuring PP and MP came to the market, the Colvard was widely used in studies with MIOLs (Table 2).

Nowadays, several devices have been proposed for measuring the pupil diameter. However, the light intensity and classification of PP and MP can vary. For instance, the VX120 (Visionix-Luneau Technologies, Chartres, France) measures pupil diameter for four light conditions from scotopic to photopic, grading as mesopic at 160 lux and photopic at 220 lux [35]. This classification is not consistent with handheld infrared pupillometers, which classified high mesopic at 6.61 lux [36], or in our study where mesopic was ~5 lux. The Sirius^®^ (SCHWIND eye-tech-solutions, Kleinostheim, Germany) also stated in its protocol that the PP diameter as 40 lux, even though the pupil diameter can be measured up to 500 lux in the dynamic mode [37,38]. The latter value is close to the 568 lux measured with a luxmeter at our premises for the K5M [11]. The Topolyzer Vario (WaveLight; Alcon, Fort Worth, TX, USA) measures photopic and mesopic pupils sizes with similar outcomes to the K5M for the mean age of the population [11,39].

Pentacam light intensity at the surface of the cornea has been reported as 4.79 × 10^−2^ mW/cm^2^, which corresponds to 327 lux and is similar to the WaveLight^®^ Oculyzer II (Alcon, Fort Worth, TX, USA) [7]. As expected, due to the lower light intensity of PW in comparison to K5M, the PP measured with PW was 0.3 mm higher in our sample and estimated between 0.23 and 0.39 in the population with 95% confidence. However, as the MP measurement depended mainly on environmental light conditions, and this was similar in both rooms (~5 lux), the agreement was almost perfect between devices. A posterior power analysis using G Power (version 3.1, available at http://www.gpower.hhu.de/) was conducted to confirm that a power of >0.8 can be obtained for the sample of 69 eyes included to detect a true difference in population means >0.1 mm with a type I error probability of 0.05 given a standard deviation of 0.5 mm and correlation between measurements of 0.8.

Table 3 shows how the mean PP described in studies with MIOLs range from 2.4 to 3.3 mm; therefore, our result of 3.06 mm is in the middle of this range and also inside of the range from 2.9 to 3.9 mm described for the Colvard in Table 2.

The agreement between the Orbscan II (Bausch & Lomb, Rochester, NY, USA) and Pentacam (Oculus Optikgeräte, Wetzlar, Germany) has also been evaluated, demonstrating a higher PP with Pentacam than with the Orbscan II [37,40]. Once again, the differences can be justified by the lower light intensity for the Orbscan II. Although the latter is reported to start at 480 lux and decrease to 28.2 lux during the scanning process [6], a value of 130 lux was measured at our center during the scanning [11]. On the other hand, the Lenstar (Haag-Streit, Bern, Switzerland) and iTrace (Tracey Technology, Houston, TX, USA) provide bigger PP diameters than Pentacam but below the 0.2 mm of mean differences in both cases [41]. These agreement results are controversial, since higher differences have been reported with iTrace (1.89 mm) [42]. Furthermore, studies with MIOLs have reported the iTrace pupil as PP or MP, leading to confusion with values close to 4 mm near MP instead of PP size [43,44]. Thus, Asena et al. [42] reported the pupil measured in mesopic light conditions (20 Lux) to explain the higher differences between Pentacam and iTrace, with the first reporting PP and the second reporting MP in this study. This is because some devices such as the iTrace and biometers such as the Anterion (Heidelberg Engineering GmbH, Heidelberg, Germany) or the IOL Master (Zeiss Meditec, Jena, Germany) provide the pupil diameter under the environmental light conditions and measurements are usually obtained in mesopic vision [45,46]. Some caution should therefore be taken when interpreting the pupil diameter relationship with visual performance measured with devices that measure pupil size in environmental light conditions. On the other hand, studies with devices that use flash lights such as the OPD-Scan III (Nidek Co., Ltd., Gamagori, Japan) and the Sirius have reported pupil diameters closer to those measured with Pentacam [47,48,49,50].

The LoAs describe the maximum deviation expected in 95% of the cases for each individual measurement using both devices. These values were 0.63 and 0.72 mm for PP and MP, respectively, which means that the differences between a measurement taken in an eye with both devices are rarely going to exceed these values. To qualify this agreement between devices, it is important to know how far these values are from the repeatability reported using these or other methods. For instance, these values are in the range of repeatability shown by handheld pupillometers under scotopic light conditions (0.64 to 1.16 mm) [36], close to the repeatability reported for the MP measured by other biometers (0.66 to 0.74 mm) [51], and slightly higher than that measured with Pentacam HR (0.55 mm). Therefore, if a difference higher than 0.3 mm is observed between devices in a single measurement, this could be mainly due to the repeatability of the pupil measurement.

From a practical point of view, it is important to understand that the PP measured under extreme photopic and mesopic conditions, such as when measured with the K5M, could differ from the conditions in which visual performance is measured. The measurement with a hand ruler in the exam lane before the testing process was in better agreement with PW than with K5M in our study, which was explained by the lower environmental light intensity (135 lux). Authors reporting results of visual performance with pupil diameter should take this into account since the lack of correlation between measured pupils with these devices and vision testing could be due to this. A limitation of our study is that these agreement results were for a sample of patients implanted with MIOLs, and the agreement might differ for other populations. Furthermore, it should be taken into account that the measurements were taken during the postoperative period, and these can differ from those obtained in the preoperative period in which a 10% larger diameter has been reported [3,7,52]. In addition, the measurement with the hand ruler was obtained with the patient looking at a stimulus located at a distance of 4 m, and around a 0.5 mm smaller pupil diameter would be expected with the patient looking at a near stimulus [53].

## 5. Conclusions

In conclusion, our study confirmed a good agreement between K5M and PW for MP but a slight correction between 0.23 and 0.39 mm (0.3 mm in our sample) should be subtracted to PW to obtain the equivalent to K5M in photopic vision. In addition, PP in our study was close to that reported in the samples of previous studies reporting PP with the Colvard and Pentacam. Independent of this agreement, surgeons should always interpret the visual performance results with the measurement of pupil diameter closer to the conditions on which it is measured, either considering light conditions or testing distance.

## Figures and Tables

**Table 1:** Descriptive statistics for pupil diameter measurement using Keratograph 5M (K5M), Pentacam AXL Wave (PW), and a ruler. The mean ± standard deviation and median [interquartile range] are shown.

Pupil	K5M	PW	Hand Ruler	*p*-Value
Photopic	2.75 ± 0.56 ^a^ 2.8 [0.6]	3.06 ± 0.54 ^b^ 2.95 [0.59]	3.17 ± 0.46 ^c^ 3 [0]	<0.0005
Mesopic	4.64 ± 0.82 4.5 [1.1]	4.68 ± 0.80 4.56 [1.03]		0.34

Paired comparisons ^a^ vs. ^b^ and ^a^ vs. ^c^, *p* < 0.0005; ^b^ vs. ^c^ *p* = 0.44. Keratograph 5M (K5M), Pentacam AXL Wave (PW).

**Table 2:** Photopic (PP) and mesopic pupil (MP) diameters, measured with Colvard device, reported in studies with intraocular lenses.

Author	Subjects	Eyes	Age	Intraocular Lens	Device	PP	MP
Gil et al. [28]	19	19	74 ± 8	ReSTOR_SN6AD2	Colvard	3.2 ± 0.6	-
Gil et al. [28]	20	20	69 ± 13	Tecnis ZKB00	Colvard	3.4 ± 0.7	-
Gil et al. [28]	20	20	73 ± 5	Tecnis ZLB00	Colvard	3.2 ± 0.7	-
Gil et al. [28]	18	18	72 ± 7	AT LISA 809M	Colvard	3.0 ± 0.6	-
Gil et al. [28]	19	19	69 ± 10	AT LISA tri 839MP	Colvard	3.3 ± 0.8	-
Gil et al. [28]	20	20	68 ± 6	Symfony ZXR00	Colvard	3.3 ± 0.8	-
Chang et al. [29]	36	72	56 ± 7	Tecnis ZMB00	Colvard	-	4.7 ± 0.8
Gil et al. [30]	12	24	63 ± 9	ReSTOR SN6AD1	Colvard	3.1 ± 0.6	4.6 ± 1.0
Gil et al. [30]	11	22	69 ± 7	Tecnis ZMA00	Colvard	3.0 ± 0.4	4.8 ± 0.4
Fernández-V-C et al. [31]	30	60	77 ± 6	Vivity DFT015	Colvard	2.9 ± 0.6	4.6 ± 0.8
Pepose et al. [32]	26	52	63 ± 6	Crystalens AO	Colvard	3.2 ± 0.6	6.1 ± 0.7
Pepose et al. [32]	25	50	64 ± 7	ReSTOR SN6AD1	Colvard	3.2 ± 0.7	6.2 ± 1.3
Pepose et al. [32]	22	44	63 ± 9	Tecnis ZMA00	Colvard	3.4 ± 0.6	6.4 ± 0.8
Alfonso et al. [33]	22	44	68 ± 6	Eyhance ICB00	Colvard	3.9 ± 0.9	5.7 ± 0.9
Fernández-V-C et al. [34]	22	22	71 ± 9	Vivity DFT015	Colvard	3.0 ± 0.5	4.7 ± 0.7

**Table 3:** Photopic (PP) and mesopic pupil (MP) diameters added, measured with Pentacam device, reported in studies with intraocular lenses.

Author	Subjects	Eyes	Age	Intraocular Lens	Device	PP
Tañá-Sanz et al. [14]	25	50	68 ± 7	Xact Mono-EDOF ME4	Pentacam	2.8 ± 0.5
Eguileor et al. [16]	15	30	72 ± 8	Eyhance ICB00	Pentacam	2.4 ± 0.3
Eguileor et al. [16]	15	30	74 ± 8	Tecnis ZCB00	Pentacam	2.5 ± 0.5
Nejat et al. [17]	23	46	58 ± 11	AT LISA tri 839MP	Pentacam	2.8 ± 0.6
Zhu et al. [18]	20	40	60 ± 7	Symfony ZXR00	Pentacam	2.7 ± 0.5
Zhu et al. [18]	21	42	59 ± 6	AT LISA tri 839MP	Pentacam	2.9 ± 0.4
Sun et al. [15]	20	20	66 ± 13	AT LISA tri 839MP	Pentacam	3.3 ± 0.6
Sun et al. [15]	20	20	71 ± 11	AT LISA tri 839MP	Pentacam	2.8 ± 0.5
Sun et al. [15]	20	20	70 ± 10	SBL-3	Pentacam	3.3 ± 0.8
Sun et al. [15]	20	20	68 ± 8	SBL-3	Pentacam	2.9 ± 0.4
Rementería-Capelo [19]	14	28	67 ± 10	PanOptix TFNT00	Pentacam	2.6 ± 0.6
Rementería-Capelo [19]	13	26	66 ± 6	RayOne Trifocal	Pentacam	3.0 ± 0.7

## Data Availability

Data available on request due to restrictions e.g., privacy or ethical.
